# A Computationally Designed Hemagglutinin Stem-Binding Protein Provides In Vivo Protection from Influenza Independent of a Host Immune Response

**DOI:** 10.1371/journal.ppat.1005409

**Published:** 2016-02-04

**Authors:** Merika Treants Koday, Jorgen Nelson, Aaron Chevalier, Michael Koday, Hannah Kalinoski, Lance Stewart, Lauren Carter, Travis Nieusma, Peter S. Lee, Andrew B. Ward, Ian A. Wilson, Ashley Dagley, Donald F. Smee, David Baker, Deborah Heydenburg Fuller

**Affiliations:** 1 Department of Microbiology, University of Washington, Seattle, Washington, United States of America; 2 Institute for Protein Design, Department of Biochemistry, University of Washington, Seattle, Washington, United States of America; 3 Washington National Primate Research Center, University of Washington, Seattle, Washington, United States of America; 4 Department of Integrative Structural and Computational Biology, The Scripps Research Institute, La Jolla, California, United States of America; 5 Skaggs Institute for Chemical Biology, The Scripps Research Institute, La Jolla, California, United States of America; 6 Institute for Antiviral Research, Department of Animal, Dairy and Veterinary Sciences, Utah State University, Logan, Utah, United States of America; 7 Howard Hughes Medical Institute, University of Washington, Seattle, Washington, United States of America; Institute of Microbiology, Chinese Academy of Sciences, CHINA

## Abstract

Broadly neutralizing antibodies targeting a highly conserved region in the hemagglutinin (HA) stem protect against influenza infection. Here, we investigate the protective efficacy of a protein (HB36.6) computationally designed to bind with high affinity to the same region in the HA stem. We show that intranasal delivery of HB36.6 affords protection in mice lethally challenged with diverse strains of influenza independent of Fc-mediated effector functions or a host antiviral immune response. This designed protein prevents infection when given as a single dose of 6.0 mg/kg up to 48 hours before viral challenge and significantly reduces disease when administered as a daily therapeutic after challenge. A single dose of 10.0 mg/kg HB36.6 administered 1-day post-challenge resulted in substantially better protection than 10 doses of oseltamivir administered twice daily for 5 days. Thus, binding of HB36.6 to the influenza HA stem region alone, independent of a host response, is sufficient to reduce viral infection and replication *in vivo*. These studies demonstrate the potential of computationally designed binding proteins as a new class of antivirals for influenza.

## Author Summary

Influenza is a major public health threat, and pandemics, such as the 2009 H1N1 outbreak, are inevitable. Due to low efficacy of seasonal flu vaccines and the increase in drug-resistant strains of influenza viruses, there is a crucial need to develop new antivirals to protect from seasonal and pandemic influenza. Recently, several broadly neutralizing antibodies have been characterized that bind to a highly conserved site on the viral hemagglutinin (HA) stem region. These antibodies are protective against a wide range of diverse influenza viruses, but their efficacy depends on a host immune effector response through the antibody Fc region (ADCC). Here we show that a small engineered protein computationally designed to bind to the same region of the HA stem as broadly neutralizing antibodies mediated protection against diverse strains of influenza in mice by a distinct mechanism that is independent of a host immune response. Protection was superior to that afforded by oseltamivir, a lead marketed antiviral. Furthermore, combination therapy with low doses of the engineered protein and oseltamivir resulted in enhanced and synergistic protection from lethal challenge. Thus, through computational protein engineering, we have designed a new antiviral with strong biopotency *in vivo* that targets a neutralizing epitope on the hemagglutinin of influenza virus and inhibits its fusion activity. These results have significant implications for the use of computational modeling to design new antivirals against influenza and other viral diseases.

## Introduction

The influenza envelope glycoprotein hemagglutinin (HA) on the surface of the influenza virus consists of a highly variable globular head domain (HA1) and a more conserved stem domain (HA2/HA1) [1, 2]. Influenza viruses comprise two phylogenetic groups (Groups 1 and 2) consisting of 18 HA subtypes and numerous genetic variants or strains within each subtype. Although vaccination can prevent influenza infection, current vaccines are strain specific, and provide minimal protection against drifted or shifted strains or subtypes [3–5]. New antivirals that broadly protect against a wide range of influenza variants are urgently needed to supplement the protective effects of vaccines and improve treatment options against seasonal influenza and future pandemics.

Broadly neutralizing monoclonal antibodies (bnAbs) that bind the conserved HA stem can neutralize diverse influenza strains *in vitro*, suggesting that antivirals targeting the HA stem could provide similar widespread protection. BnAbs bind to the fusogenic region of the HA stem and inhibit the conformational rearrangements in HA required for membrane fusion [6–8]. Recent studies show that protection by HA-stem binding bnAbs is greatly enhanced through FcγR engagement *in vivo* [1, 9]. While antibody binding to the fusogenic region is sufficient for *in vitro* neutralization of the virus, Fc-FcγR interaction and activation of antibody-dependent cellular cytotoxicity (ADCC) are critical for *in vivo* efficacy of stem-binding bnAbs [1, 10].

We previously described two computationally designed small proteins that bind the HA stem region of multiple Group 1 influenza virus HA subtypes with equal or higher affinity than most bnAbs [11, 12]. These results demonstrated the feasibility of using computational modeling to design a protein that mimics the stem binding of bnAbs in vitro, but since the designed proteins lacked an Fc, it was unclear if they would be able to afford protection against a rigorous influenza challenge in vivo. Here, we optimized one of these HA stem binding protein for tighter binding using deep mutational scanning [13] and investigated its ability to afford protection against influenza infection *in vivo*. We show that intranasal administration of an HA stem binding protein reduces viral replication and provides strong protection against diverse influenza strains when administered as a prophylactic or therapeutic *in vivo*. We further show that protection is independent of the host immune response, demonstrating that an antiviral can disrupts influenza infection in vivo via direct binding to the HA stem.

## Results

### HA stem-binding protein affords prophylactic and therapeutic protection against influenza in vivo

**Fig 1:**
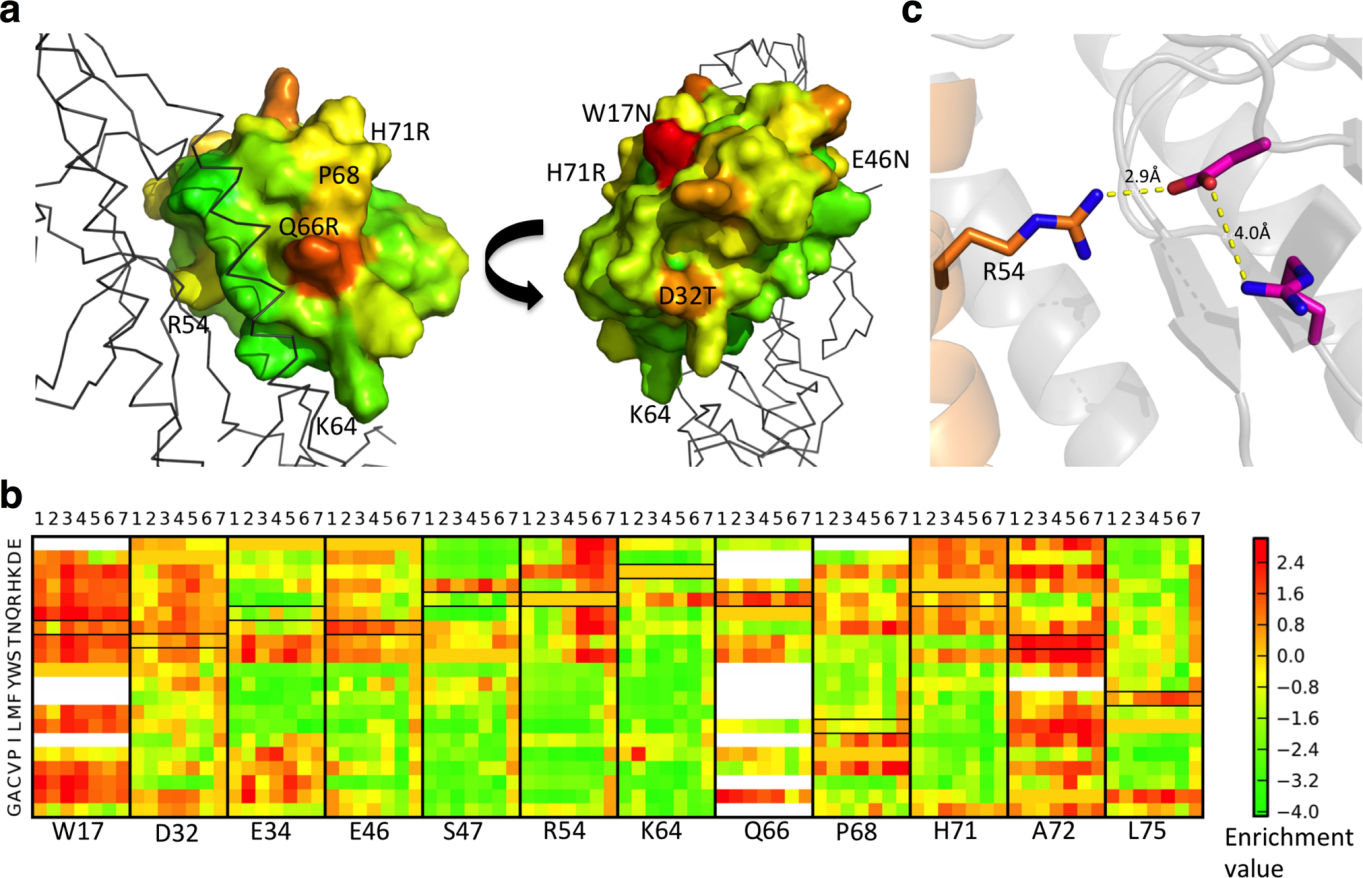
Specificity-enhancing mutations and overall affinity-increasing substitutions using selection against different HA subtypes. (**a**) HB36.5, in complex with HA, colored by average residue enrichment in FACS sorts against 7 different HA subtypes. Final residue identity after mutagenesis and selection to obtain HB36.6 are labeled in black. Positions 54, 64, and 68 retained their HB36.5 identities. (**b**) Enrichment of substitutions at 12 key positions in HB36.5 in selections against each HA strain. Labels at the bottom indicate position in HB36.5; numbers at the top represent the different flu strains and subtypes (1: A/South Carolina/1/1918 (H1), 2: A/California/04/2009 (H1), 3: A/Vietnam/1203/2004 (H5), 4: A/Indonesia/05/2005 (H5), 5: A/Adachi/2/1957 (H2), 6: A/turkey/Wisconsin/1/1966 (H9), 7: A/duck/Alberta/60/1976 (H12)). At most positions, the enrichment profiles against the different HA strains are similar but, at several positions, they are quite distinct. At position 54, for example, arginine is highly conserved and substitutable for lysine in selections for binding against HAs 1–4, but is outcompeted by smaller charged/polar residues in selections against HAs 5–7 (red region at upper right of R54 panel). White cells indicate insufficient data (<15 sequences in the input library) and black boxes indicate the residue identities in HB36.6. (**c**) Origin of HA strain dependence of substitutions at HB36.5 position R54. Arg54 forms a hydrogen bond network with Asp and Arg residues in HAs 1–4. In HAs 5–7, the Asp is substituted by a Glu, disrupting the interface with Arg54 leading to a preference for smaller polar residues.

**Fig 2:**
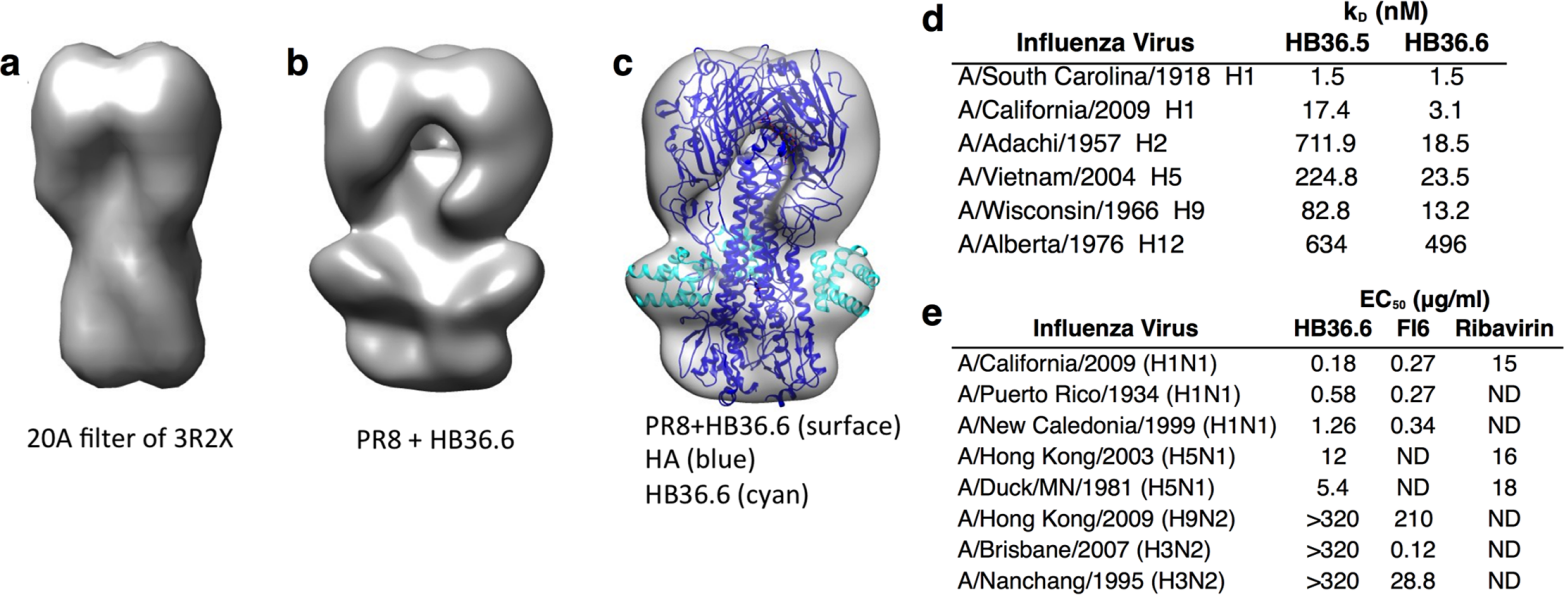
Characterization of HB36.6. (a) Crystal structure of A/South Carolina/1/1918 (H1) HA (derived from PDB 3R2X) filtered to 20 Å resolution. (b) Negative-stain EM reconstruction of PR8 HA bound to HB36.6. (c) X-ray structure of A/South Carolina/1/1918 (H1) HA (PDB 3R2X) (blue) in complex with HB36.6 (cyan) docked into PR8 EM reconstruction in b. HB36.6 fits well into the extra density in the stem region. (d) Equilibrium binding constants determined by biolayer interferometry for HB36.5 and HB36.6 against six HAs demonstrate broad improvements against a variety of Group 1 subtypes. (e) EC_50_ (μg/ml), compound concentration that reduces viral replication by 50%, of HB36.6, the monoclonal antibody FI6v3, and ribavirin against five representative strains from H1, H9, and H5 Group 1 viruses and two H3 Group 2 viruses. ND: not determined.

We optimized a broadly cross-reactive HA binding protein, HB36.5, which is a stable, 97-residue designed protein, by increasing its affinity against multiple HA subtypes [12]. We constructed a library in which each residue was individually mutated to all other possible amino acids and carried out two rounds of yeast surface display selection against seven different Group 1 HA subtypes. We then sequenced the libraries and computed the enrichment (or depletion) of each individual point mutant during affinity maturation. The core of the binding interface was highly conserved in the selections against different HA subtypes (**Fig 1A, green**), but several mutations in HB36 in the second shell of residues close to the binding interface were enriched across all seven subtypes (**Fig 1A (red) and 1B**). Multiple subtype-specific substitutions were also identified around the periphery of the binding interface (**Fig 1B**), which reflect changes to accommodate HA sequence differences near the interface (**Fig 1C**). We made a combinatorial library of substitutions that were enriched across all subtype selections at 12 mutated positions and carried out three rounds of yeast display sorting against A/South Carolina/1/1918 (H1N1) HA, which converged on a variant with nine substitutions called HB36.6. Negative-stain electron microscopy revealed that HB36.6 binds to the designed target location on the HA (**Fig 2A, 2B and 2C**). Biolayer interferometry showed that HB36.6 had higher affinity than HB36.5 against a wide range of Group 1 influenza subtypes, with greater than 40-fold and 10-fold affinity increases against H2 and H5 HAs, respectively (**Fig 2D and S1 Table**).

*In vitro*, HB36.6 potently neutralized a panel of genetically distinct human (H1N1) and avian (H5N1) influenza viruses (range of genetic diversity between HA amino-acid sequences is 50–89%) with a 50% effective concentration (EC_50_) range of 0.18–12.0 μg/ml (**Fig 2E**). This level is comparable to the EC_50_ range of 0.27–0.34 μg/ml observed for the monoclonal antibody, FI6v3, which has been shown to broadly neutralize Group 1 and 2 influenza strains [6]. In addition, HB36.6 was more potent than ribavirin, a broad-spectrum antiviral that neutralizes influenza *in vitro* [14–16] and protects against influenza in mice [15, 17] but had a higher EC_50_ of 15–18 μg/ml against a representative subset of the same influenza strains (**Fig 2E**). However, HB36.6 did not neutralize either of the Group 2 strains tested or a Group 1 A/Hong Kong/2009 H9N2 strain, results that are consistent with computationally designed stem binders not binding Group 2 viruses [11] and FI6v3 weakly neutralizing the same H9N2 virus with an EC_50_ of 210 μg/ml.

**Fig 3:**
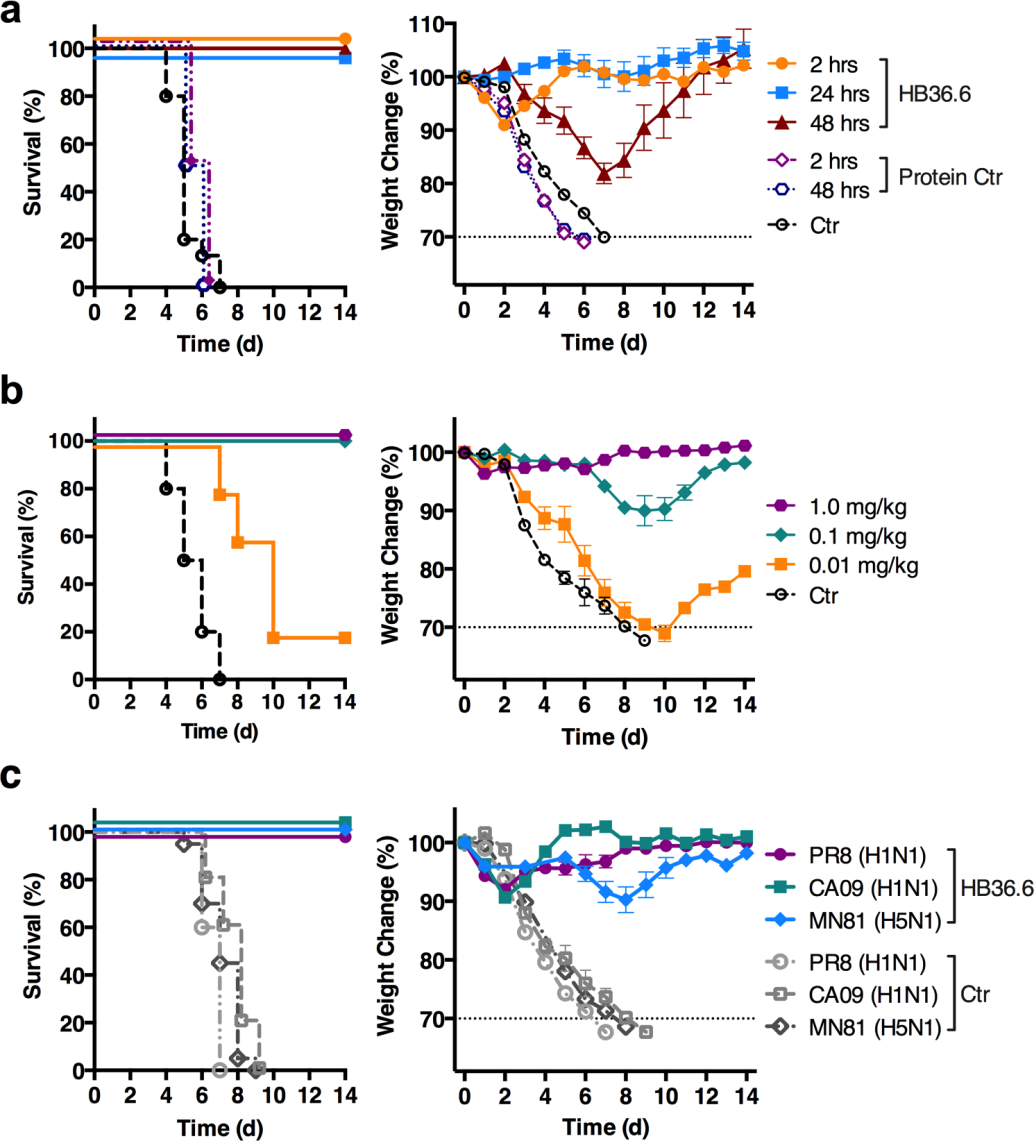
Intranasal delivery of HB36.6 affords prophylactic protection against lethal challenge by influenza virus. (a) Survival and weight change in BALB/c mice (n = 10 per group) that received 6.0 mg/kg of HB36.6 administered intranasally (IN) at 2, 24, or 48 hours before challenge with 10 MLD_50_ CA09 virus. The Protein Control (Ctr) group received 6.0 mg/kg of lysozyme at 2 or 48 hours before challenge with 10 MLD_50_ CA09 virus. (b) Survival and weight change in BALB/c mice (n = 5 per group) that received 0.01–1 mg/kg IN doses of HB36.6 2 hours before challenge with 10 MLD_50_ of CA09 virus. (c) Survival and weight change in BALB/c mice (n = 10 per group) that received 3.0 mg/kg of HB36.6 IN 2 hours before IN infection with 10 MLD_50_ of H1N1 CA09 virus, 6 MLD_50_ H1N1 A/PR/8/34 (PR8), or 3 MLD_50_ of H5N1 A/Duck/MN/1525/81 (MN81) virus. Mean and SEM are shown.

We next investigated the ability of HB36.6 to protect against influenza in mice. We administered a single intranasal (IN) dose of HB36.6 (6.0 mg/kg) to BALB/c mice at 2, 24, or 48 hours prior to challenge with a lethal dose (10 times the 50% mouse lethal dose or 10 MLD_50_) of H1N1 A/California/04/2009 (CA09) virus. CA09 is a highly virulent Group 1 pandemic influenza strain that leads to rapid weight loss and death in mice within 5–8 days post-infection (d.p.i.) [18]. When administered up to 48 hours before challenge, a single pre-exposure dose of HB36.6 afforded complete protection with 100% survival and little (<10%) to no weight loss, whereas all untreated controls (Ctr) exhibited >30% weight loss and no survival (**Fig 3A**). Protection was specific to HB36.6 since a protein control (lysozyme, 6.0 mg/kg), administered either 48 or 2 hours before CA09 challenge provided no protection and resulted in weight loss and mortality comparable to the controls (**Fig 3A**). Protection was dependent on the IN route of delivery because the same dose of HB36.6 delivered intravenously (IV) provided no protection (**S1 Fig**). When administered intranasally, HB36.6 was readily detected throughout the lung within 6 hours after administration indicating penetration into the lower respiratory tract (**S2 Fig**). The observed prophylactic protection between 48–72 hours before challenge suggests a bioavailability range within this timeframe, although additional studies to determine the bioavailability and pharmacokinetics of HB36.6 will be needed.

Lower doses of 1.0, 0.1, and 0.01 mg/kg administered IN two hours prior to lethal challenge with CA09 also resulted in 100% survival with little (0.1 mg/kg) or no (1.0 mg/kg) weight loss whereas controls exhibited rapid weight loss and succumbed to the infection within 7 d.p.i. (**Fig 3B**). Mice that received the lowest IN dose tested (0.01 mg/kg) exhibited weight loss, yet survived 2–3 days longer than controls and 20% of mice completely recovered.

To determine if HB36.6 can protect against genetically distinct strains *in vivo*, we investigated protection against H1 and H5 viruses that are the most virulent Group 1 subtypes that infect mice and cause the majority or most severe Group 1 influenza infections in humans. We inoculated mice IN with HB36.6 (3.0 mg/kg) two hours before challenge with either CA09, A/PR8/34 (PR8), another highly virulent H1N1 mouse-adapted virus (PR8), or the H5N1 avian strain A/Duck/MN/1525/81 (MN81). The HA protein sequence of CA09 is 18% and 36% divergent from PR8 and MN81, respectively. HB36.6 provided robust protection against these two genetically distinct H1N1 viruses and the highly pathogenic H5N1 virus, with 100% of the mice surviving and no weight loss (**Fig 3C)**. This result is consistent with the *in vitro* results showing that HB36.6 broadly binds and neutralizes H1 and H5 HAs (**Fig 2D and 2E**).

**Fig 4:**
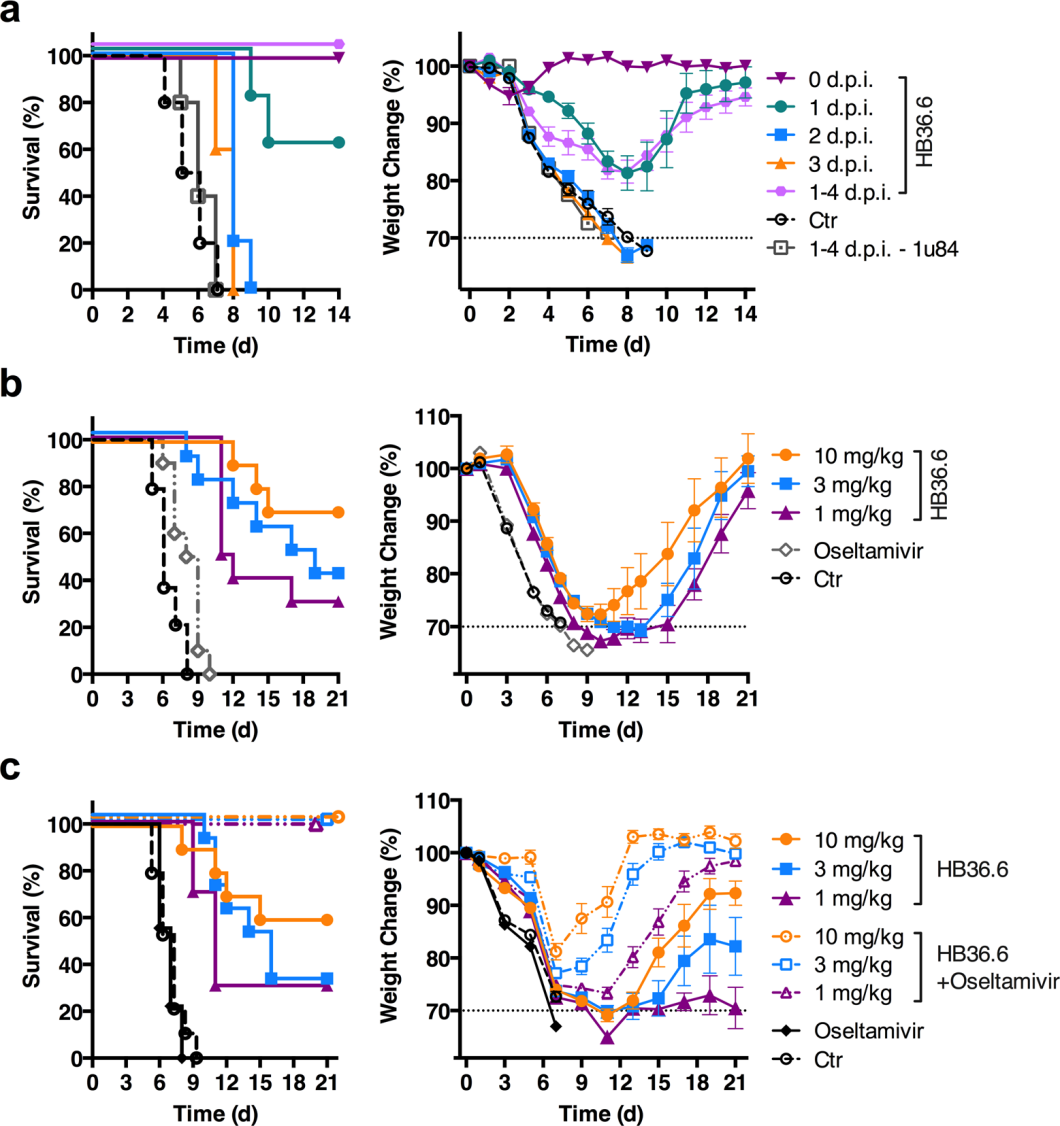
Intranasal delivery of HB36.6 affords therapeutic protection against lethal challenge by influenza virus. (a) Survival and weight change in BALB/c mice (n = 10 per group) that received 3.0 mg/kg of HB36.6 IN on day 0 (2 hours post-infection) or +1, +2, or +3 days post-infection (d.p.i.) or once daily on days +1–4 post-infection with 10 MLD_50_ CA09 virus. As a control, the scaffold protein 1u84 (3.0 mg/kg) was IN administered once daily on days +1–4 post-infection. (b) Survival and weight change in BALB/c mice (n = 10 per group) that were challenged with 3 MLD_50_ of CA09 virus and then received either a single dose of HB36.6 (0.1–10 mg/kg) IN on day +1 p.i., or oseltamivir (2.5 mg/kg/dose) by oral gavage twice a day on days +1–5 p.i. (10 doses total). Mean and SEM are shown. (c) Survival and weight change in BALB/c mice (n = 10 per group) that were challenged with 3 MLD_50_ of CA09 virus and then received either a single dose of HB36.6 (1–10 mg/kg) IN on day +1 p.i., Oseltamivir (2.5 mg/kg/dose) by oral gavage twice a day on days +1–5 p.i. (10 doses total), or a combination of HB36.6 (1–10 mg/kg) by IN on day +1 plus oseltamivir (2.5 mg/kg/dose) by oral gavage twice a day on days +1–5 p.i. (10 doses total). Mean and SEM are shown.

We next investigated HB36.6 for the ability to protect post-exposure. We challenged mice with CA09 and then treated with either a single IN dose of 3.0 mg/kg HB36.6 on day 0 (2 hours p.i.), +1, +2 or +3 p.i. or four daily IN doses on days +1–4 p.i. HB36.6 reduced weight loss and afforded complete recovery and protection from lethality in 100% of mice when administered daily for 4 days or as a single inoculation 2 hours p.i. and 60% protection from lethality when administered as a single inoculation + 1 day p.i. (**Fig 4A**). There was no significant difference in weight loss between mice receiving daily doses on days +1–4 p.i. or a single dose at 2 hours or day +1 p.i., suggesting that a single dose within 1 day post-exposure is sufficient to protect from disease. Although mice that received HB36.6 at day +2 or +3 p.i. succumbed to infection, disease onset was delayed. The majority of these mice died at 8–9 d.p.i., whereas 100% of the controls succumbed within 4–7 d.p.i. (2 d.p.i., p = 0.0006; 3 d.p.i, p = 0.0031 compared to controls) (**Fig 4A**). The protection was specific for HB36.6 binding to the HA since daily administration of the scaffold protein (PDB ID 1u84) that HB36.6 was modeled on provided no protection (**Fig 4A**).

We next compared a single dose of HB36.6 to oseltamivir [19], an antiviral that targets influenza neuraminidase and is currently used to treat influenza in humans. We challenged mice with CA09 virus and then treated with either a single IN dose of HB36.6 (1.0–10 mg/kg) on day +1 p.i., or the recommended schedule of ten doses of oseltamivir administered by oral gavage, twice daily for 5 days starting on day +1 p.i.(5 mg/kg/day) [17, 20, 21]. Oseltamivir afforded a modest delay in time to death, but provided no protection (0%) from lethality. In contrast, escalating doses of HB36.6 protected mice from lethality with the highest dose (10 mg/kg) affording 70% efficacy (**Fig 4B**). Thus, a single dose of HB36.6 provided superior protection against a highly virulent influenza challenge when compared to ten doses (2 times per day for 5 days) of a leading influenza antiviral. Furthermore, post-infection treatment with a combination of a sub-optimal single dose of HB36.6 (1–10 mg/kg) and twice-daily doses of Oseltamivir resulted in 100% survival, indicating a synergistic effect when the two antivirals are combined (**Fig 4C**).

### HB36.6 reduces viral load and inflammation

**Fig 5:**
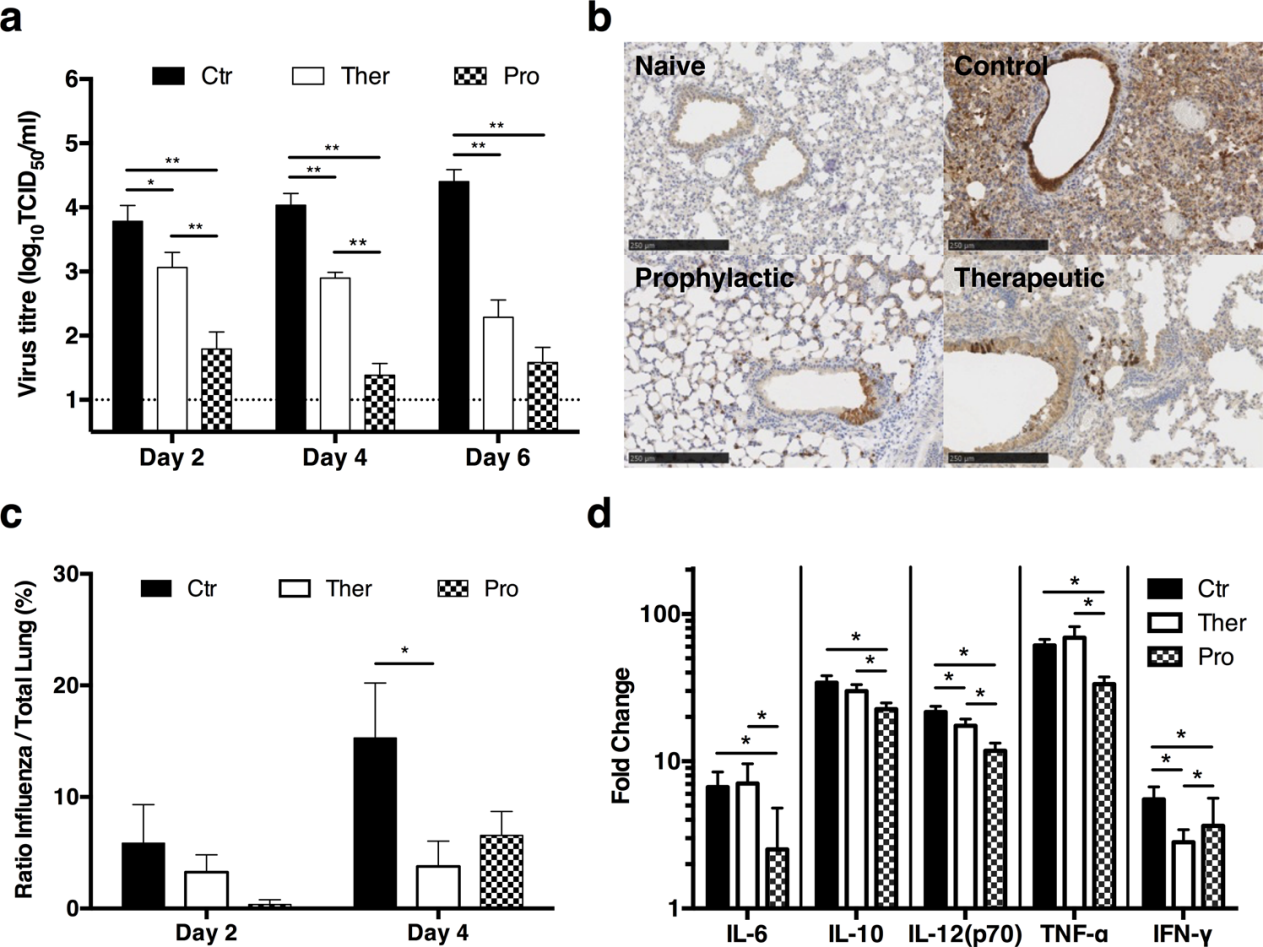
HB36.6 suppresses viral replication and inflammation in the lung. (a) Viral titers in nasal washes of untreated infected controls (Ctr) and mice that received 6.0 mg/kg HB36.6 either 1 day before (Prophylaxis, Pro) or 1 day after (Therapeutic, Ther) infection with 10 MLD_50_ CA09 virus. Nasal washes collected on days 2, 4 and 6 post-infection were measured by determining the 50% tissue culture infectious dose (TCID_50_) (bars indicate mean viral titer ±SD, n = 18 mice per group, three replicate experiments). (b) IHC staining of intracellular influenza NP (H1N1) of representative lung sections from uninfected (Naïve) and untreated infected controls (Control) and HB36.6-treated mice (Prophylactic and Therapeutic) at 4 days post-infection with 10 MLD_50_ CA09 virus. Mouse lungs were not inflated with formalin and consequently resulted in lung collapse and a more hypercellular appearance in the uninfected control. Images selected show representative staining of influenza (NP) positive cells for each group. (c) Quantification of influenza positive cells in lung tissues was performed by measuring the area of positive staining compared to the total tissue on the slide (uniform random sampling of 50% lung tissue). (d) Inflammatory cytokines were assayed by Bio-Plex using supernatants from lung homogenates obtained from BALB/c mice on day 2 following infection with 10 MLD_50_ CA09 virus (n = 8 mice per group). The fold change over naïve-uninfected mice is shown. For a, c and d, significant differences between the Pro and Ther groups to the Ctr group are shown: *P < 0.05, **P < 0.001.

To determine the effects of HB36.6 at the respiratory sites of virus exposure, we analyzed viral replication in nasal and lung compartments in mice that received a single IN dose of HB36.6 (6.0 mg/kg) either 24 hours before (Prophylactic- Pro) or after (Therapeutic- Ther) challenge with CA09. We collected nasal washes on days 2, 4, and 6 post-challenge and viral titers were measured by an end-point dilution assay (TCID_50_). At each time-point p.i., mice that were treated with HB36.6 before (Pro) or after (Ther) challenge exhibited a substantial 1–3 log-fold reduction in mean viral titer when compared to untreated controls, with the lowest viral load observed in the prophylactic group (**Fig 5A**).

We next investigated the effects of HB36.6 on viral replication in the lung. We treated mice IN with HB36.6 (6.0 mg/kg) 1 day before or post-infection with CA09, collected lung tissue on days 2 and 4, and then stained for intracellular expressed influenza nucleoprotein (NP) to identify infected cells. Lung tissues from mice that received prophylactic or therapeutic administration of HB36.6 showed less viral replication in the lungs when compared to the untreated controls at day 4 p.i. (**Fig 5B**) and *in situ* enumeration of NP positive cells in the lung tissue confirmed significantly lower numbers of infected cells in the lung at day 4 in mice that received HB36.6 as a therapeutic compared to controls (P ≤ 0.0263, **Fig 5C**). The lower nasal wash viral loads in the prophylactic group (**Fig 5A**) but comparable lung viral loads in the prophylactic and therapeutic groups (**Fig 5B and 5C**) suggest that prophylaxis with HB36.6 likely affords protection by binding and blocking the virus at the nasal site of exposure, whereas post-exposure therapy with HB36.6 affords protection by containing the burst of viral replication and progeny release in the lung resulting in reduction of viral load in the lung and blunting of the inflammatory response that typically initiates within 24 hours after challenge [18].

Influenza infection results in the expression of cytokines that induce inflammation and recruit activated immune cells to clear the infection. However, this inflammatory response damages the pulmonary epithelium and increases susceptibility to secondary infections by ~100 fold [22, 23]. To determine if HB36.6 protects from influenza-induced inflammation, mice were administered a single IN dose of HB36.6 (6.0 mg/kg) either 24 hours before (Pro) or 24 hours after (Ther) lethal challenge with CA09. Lungs were collected on day 2 p.i. and supernatants from lung homogenates were analyzed for the expression of inflammatory cytokines (IL-6, IL-10, IL-12(p70), TNF-α, IFN-γ). HB36.6 delivered as a prophylactic significantly lowered several cytokines, including the inflammatory cytokines IL-6 and TNF-α, when compared to controls (P≤0.0012, **Fig 5D**). HB36.6 delivered as a therapeutic also significantly lowered the amount of IL-12(p70) and IFN-γ when compared to controls (P≤0.0007, **Fig 5D**). These results suggest that reduction in viral load by HB36.6 provided an additional benefit of decreasing the cytokine responses that typically lead to increased inflammation and tissue damage. Together, these results show that HB36.6 blocks and interferes with viral spread, resulting in a lower viral replication, suppression of the cytokine response, and decreased lung inflammation. Furthermore, since HB36.6 lacks an Fc domain, these results show that engagement of the host FcγR is not required for protection in vivo.

### HB36.6 does not induce a protective host antiviral response

**Fig 6:**
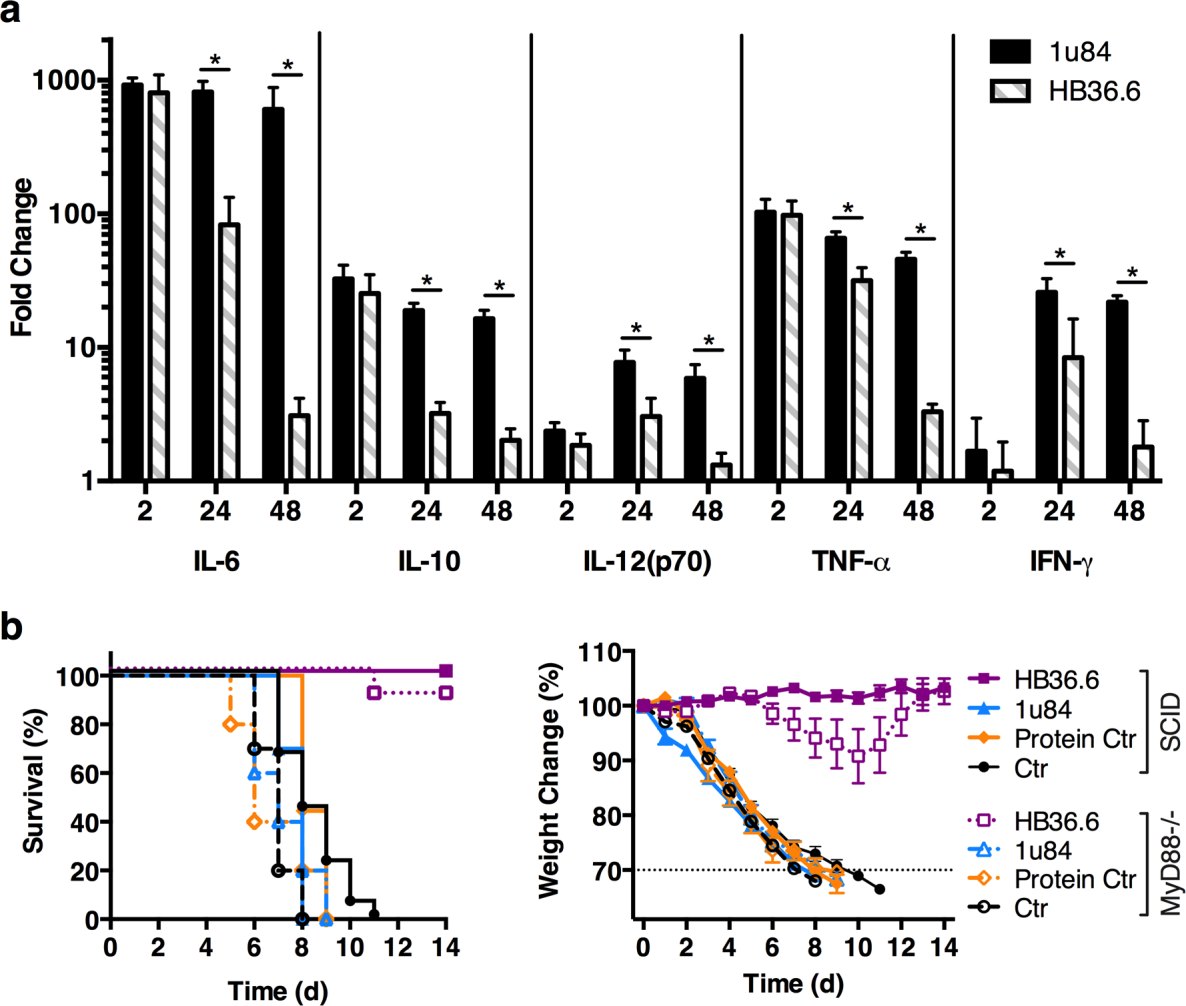
HB36.6 induces a transient cytokine response that is not required for protection. (a) Inflammatory cytokines were assayed by Bio-Plex using supernatants from lung homogenates obtained from BALB/c mice 2, 24 and 48 hours following administration with HB36.6 (6.0 mg/kg) or the scaffold protein (PDB ID 1u84) (6.0 mg/kg) (n = 10 mice per group). Fold change over naïve mice is shown. *P < 0.05. (b) Survival and weight change in SCID and MyD88-/- mice (n = 10 per group) that received 6.0 mg/kg of HB36.6, scaffold protein, or Protein Ctr (lysozyme) IN 2 hours before IN infection with 10 MLD_50_ CA09 virus.

Small proteins, such as HB36.6, may stimulate an immune response that could interfere with the effectiveness of a second administration or alternatively, stimulate antiviral responses that can contribute to protection [24]. Four doses of HB36.6 administered 2 weeks apart induced very low levels of antibody; however, 100% of mice were still completely protected when challenged with a lethal dose of CA09 1 day after the 4^th^ dose (**S3 Fig**). These results indicate that HB36.6 is poorly immunogenic and repeat administration does not interfere with the antiviral activity of subsequent doses. However, induction of even a modest antibody response after multiple doses suggested HB36.6 likely stimulated a host innate response. To determine if HB36.6 administration induces antiviral cytokine responses that could contribute to protection, cytokines were measured at different time-points post-HB36.6 administration. Mice either received a single IN dose of HB36.6 (6.0 mg/kg) or the scaffold protein (PDB 1u84, 6.0 mg/kg) and lungs were collected at 2, 24 or 48 hrs post-administration. Supernatants from lung homogenates were analyzed for the expression of inflammatory cytokines (IL-6, IL-10, IL-12(p70), TNF-α, IFN-γ). Both HB36.6 and scaffold protein induced low levels of cytokines that peaked between 2–24 hrs post-administration and, by 48 hrs, the levels had dropped to pre-administration levels (**Fig 6A**). Importantly, cytokine levels after HB36.6 administration were significantly lower than levels induced by scaffold protein that afforded no protection from challenge. These data suggest that, although administration with HB36.6 induced a low cytokine response, the levels were too transient and/or low to contribute to protection.

To investigate the possibility that HB36.6 may induce other host responses that could contribute to protection, we tested HB36.6 for protection against influenza in two severe immune-deficient mouse models: NOD SCID gamma (SCID) and MyD88-/- mice. SCID mice lack mature T, B, and NK cells and are unable to develop an adaptive immune response [25, 26]. MyD88-/- mice lack TLR signaling and are deficient in cytokine signaling, resulting in a severely dampened innate and adaptive immune response [27–29]. HB36.6 (6.0 mg/kg), scaffold protein (6.0 mg/kg), and another protein control (lysozyme, 6.0 mg/kg) were IN administered 2 hours before challenge with CA09. HB36.6 protected 100% of the SCID mice and 90% of the MyD88-/- mice with only minimal weight loss (**Fig 6B**), whereas the control SCID and MyD88-/- mice (1u84, Protein, and Naïve) exhibited significant weight loss and 0% survival. These results provide further evidence that the antiviral effect of HB36.6 is likely due to direct binding to the HA stem and is independent of an antiviral host response.

## Discussion

We showed previously that computationally designed proteins optimized for high affinity binding to a viral protein can neutralize viruses in vitro [11, 12]. However, prior to this study, it was not known if such proteins would have sufficient stability and potency to afford protection in vivo. Here, we provide the first proof-of-concept that a novel small protein that was computationally designed to mimic bnAbs and bind the highly conserved HA stem could be developed into a highly effective antiviral capable of neutralizing and affording robust protection against diverse strains of influenza *in vivo*. We show that HB36.6 neutralizes a panel of Group 1 H1 and H5 viruses in vitro and a single intranasal dose afforded significant protection against three highly divergent H1 and H5 influenza strains *in vivo*. This suggests that the range of neutralizing specificity of HB36.6 observed *in vitro* translated to protection against these strains *in vivo*.

Our studies also show that HB36.6 mediates protection independent of a host response. This contrasts to previous studies employing intravenous injection of a bnAb (FI6v3) that HB36.6 binding was designed to mimic. These studies showed that engagement with the host’s FcγR and recruitment of ADCC was crucial for optimum protection by bnAb in vivo [1, 9]. Here, we show that HB36.6, which lacks an Fc, still affords robust protection against different strains of influenza *in vivo*. This outcome may be due to intranasal delivery of HB36.6, which localizes the antiviral at the respiratory site of viral exposure and/or the ability of HB36.6 to bind the stem with high affinity [30]. Consistent with this possibility, Leyva-Grado et.al [30] showed that intranasal delivery of the fragment antigen-binding (Fab) region from the broadly neutralizing antibody, FI6v3, afforded a similar degree of protection as we report here for HB36.6.

A recent study showed that a host receptor binding peptide provided prophylactic protection against lethal influenza challenge that depended on the induction of an inflammatory antiviral response [24]. The peptide did not work as a therapeutic, since antiviral cytokines are less effective after a viral infection is already established. In contrast, we found that HB36.6 induced only weak cytokine responses that were lower than the non-protective scaffold protein control and provided protection in two severe immune-deficient mouse models indicating a mechanism that is independent of a host antiviral cytokine immune response. Together, these results indicate that binding to the HA stem alone was sufficient for in vivo protection against influenza. These findings have implications for development of HB36.6 as a safe and effective alternative for protection from influenza. Here, we found that pre-exposure treatment with HB36.6 prevented infection without inducing an inflammatory response, hence it could be used pre-exposure to increase resistance to infection without the risk of inducing adverse inflammatory responses. Furthermore, since post-exposure inflammation mediates enhanced influenza disease and increased susceptibility to secondary infections [22], this also suggests HB36.6 could be used to treat influenza without the risk of exacerbating disease due to immune effector-mediated inflammation. Finally, the ability of HB36.6 to mediate protection independent of the host response has further implications for protection in the immune-compromised or elderly, who comprise the majority of deaths from seasonal influenza each year [31].

The CA09 strain used in our therapeutic challenge studies is highly virulent, rapidly disseminating into the lower lung of mice within hours after challenge and causing death in control mice within 8 days [18]. Although weight loss is not seen until later time-points, the robust inflammatory response responsible for these symptoms is initiated within hours after challenge [32]. The level of protection afforded by HB36.6 against this strain when used as a therapeutic suggests significant potential to provide post-exposure benefit and improve treatment of influenza infection when compared to current treatments. Consistent with this possibility, we show that a single dose of HB36.6 administered to mice challenged with a highly virulent influenza strain outperformed a five-day, ten-dose regimen of oseltamivir, the lead antiviral approved for treatment of influenza in humans. This result is consistent with previously reported results showing that oseltamivir delayed, but did not protect from mortality in mice [33–35]. Furthermore combining sub-optimal doses of HB36.6 and oseltamivir resulted in synergistic protection, a result that suggests potential for use of HB36.6 as an approach to augment the effectiveness of existing marketed antivirals. Indeed, several studies have shown that therapeutic use of influenza antiviral combinations could increase antiviral potency, clinical effectiveness, and reduce resistance emergence [36, 37]. Although this HA stem epitope is highly conserved the potential for emergence of resistance to HB36.6 will require further investigation.

Previous studies with bnAbs, small molecule inhibitors, and proteins designed to bind the HA stem demonstrate that targeting the HA stem affords protection by inhibiting the low pH-induced fusion of the viral membrane with the endosomal membrane [1, 9, 11, 12, 38]. The direct binding of HB36.6 to the highly conserved fusion region similarly inhibits key conformational rearrangements in the HA that drive the fusion of the viral and endosomal membranes, blocking entry of the viral RNA into the cell via the endosome [11, 12]. Intravenous delivery of bnAbs has been shown to be highly effective in mice and ferrets and is being developed for the hospital setting to treat severe and complicated influenza [1, 6, 30, 39]. However, due to the route of delivery and the high cost of monoclonal antibodies, this strategy is not viable for treatment of uncomplicated influenza in the general population. An antiviral, such as HB36.6, that is effective intranasally could be more widely self-administered in the general population pre- or post-exposure to prevent infection or shorten recovery from the infection.

Seasonal drifted strains reduce vaccine efficacy and drug-resistant strains hinder the use of current antivirals in the prevention and treatment of influenza. These problems highlight the need for effective new antiviral drugs [40, 41]. Overall, our results show that computationally designed proteins have potent anti-viral efficacy *in vivo* and suggests promise for development of a new class of HA stem-targeted antivirals for both therapeutic and prophylactic protection against seasonal and emerging strains of influenza.

## Methods

### Yeast display k_D_ titrations

Wild type HB36.5 and the transformed HB36.5 site-saturation mutagenesis (SSM) yeast display library was inoculated into 1mL of synthetic dextrose casamino acids (SDCAA) medium supplemented with carbenicillin and chloramphenicol and grown overnight at 30°C, 250rpm. Cells were pelleted by centrifugation, resuspended in 200μL of synthetic galactose casamino acids (SGCAA), 40μL of the resuspended cells were inoculated into 960 additional μL of complete SGCAA and induced ~24h at 18°C, 250rpm. Cells were collected by centrifugation, washed with Phosphate Buffered Saline (PBS), 0.1% w/v bovine serum albumin (BSA) (PBSF), and diluted to optical density 600nm (OD_600_) of 2.0. 1.5x10^5^ cells were mixed with purified biotinylated hemagglutinin (HA) in PBSF individually at a range of concentrations spanning the construct’s predicted k_D_ and incubated at 22°C for 30m. After labeling with HA, the cells were collected by centrifugation, washed once with PBSF, and incubated with 0.6μL of FITC-labeled anti-CMyc antibody and 0.25μL phycoerythrin (PE)-labeled streptavidin on ice for 10m. Cells were collected, washed with PBSF, and resuspended into 200μL of PBSF. Fluorescence of 50,000 cells from each titration point was measured on an Accuri C6 flow cytometer with a 488nm laser for excitation and a 575nm band pass filter for emission. Negative controls for binding were induced cells with no HA labeling. BD Cflow software was used to measure the total PE fluorescence of the displaying cell population and a custom MATLAB non-linear curve fitting script was used to derive equilibrium binding constants for each hemagglutinin subtype.

### Site-saturation mutagenesis library construction

HB36.5 in pETCON plasmid was mutagenized individually via Kunkel’s method [42] in 86 consecutive codon positions using NNK degenerate primers purchased from Integrated DNA Technologies (Coralville, IA). Primers were designed using Firnberg’s method. The theoretical library size of all 86 reactions was 1720 amino-acid sequences. Kunkel’s reactions were purified with QiaQuick columns (Qiagen, Hilden, Germany) and pooled in groups of 12 (codon positions 1–12, 13–24, 25–36, 37–48, 49–60, 61–72, 73–86). 1μL of each plasmid pool was transformed by electroporation into XL10 Gold electrocompetent cells (Stratagene, La Jolla, CA) with a minimum efficiency of 3x10^5^ Colony Forming Units (CFUs) per pool (>100-fold coverage). Liquid cultures were grown overnight in terrific broth (TB) and harvested using a QiaPrep miniprep kit (Qiagen, Hilden, Germany). Mutated genes were amplified from each plasmid pool by adding 1μL of plasmid to 10μL of 5x Phusion Buffer, 1μL of 10mM dNTPs, 2.5μL of 10μM upGS primer, 2.5μL of 10μM downCMyc primer, and 0.5μL of Phusion polymerase in 50μL total volume. The reaction used 30 cycles of PCR (98°C 10s, 65°C 15s, 72°C 15s). PCR product was purified with a QiaQuick kit and transformed into EBY100 *S*. *cerevisiae* using Chao’s method [43] along with gel-purified pETCON vector digested with *NdeI*/*XhoI* (NEB, Waltham, MA).

### Yeast display HA selections

A transformed HB36.5 SSM yeast display library was sorted in two rounds. For each round, cells were grown in 10mL of SDCAA overnight at 30°C, collected by centrifugation, and induced in SGCAA at 18°C for ~24 hours. Cells were collected by centrifugation, washed with PBSF, and ~4x10^6^ cells labeled with purified biotinylated HA at a concentration half of the k_D_ determined by yeast display titrations or, if no k_D_ could be determined, at 500nM. In the first round of sorting, primary labeling proceeded for 30m at 22°C as described for titrations. In the second round, to select for mutations that improved binding but did not destabilize the protein, primary labeling was performed for 30m at 37°C. Secondary labeling was done with 1.2μL anti-c-Myc tag antibody conjugated to fluorescein isothiocyanate (FITC) and 0.5μL streptavidin-phycoerythrin (SAPE) in a total volume of 100μL PBSF on ice for 10m. In each sorted population, the top ~5% of FITC-displaying cells were collected.

### Illumina sequencing

Plasmid DNA was prepared as previously described [11]. Genes were amplified from the plasmid by adding 36.5μL of purified plasmid to 10μL of 5x Phusion master mix, 1μL each of pETCON_inner_fwd and pETCON_inner_rev, 1μL of 10mM dNTPs, and 0.5μL of Phusion polymerase (Thermo). The reaction used 30 cycles of PCR (98°C 10s, 58°C 15s, 72°C 15s). Correctly sized products were gel extracted using a Qiaquick gel extraction kit (Qiagen). 10μL of gel extracted reaction product were added to 10μL of 5x Phusion master mix, 1μL each of miseq_outer_fwd and miseq_outer_rev with the correct library barcode, 1μL of 10mM dNTPs, and 0.5μL of Phusion polymerase in 50μL, and amplified again with 30 cycles of PCR (98°C 10s, 58°C 15s, 72°C 15s). The two primer sets have overhangs that add Illumina sequencing primer binding sites, barcode sequences, and flow cell adaptors to the gene to be sequenced. They additionally add 12 entirely degenerate bases at the beginning of the forward and reverse read, ensuring adequate diversity for the Illumina basecalling algorithms. This enabled the DNA pools to be prepared and sequenced in two runs of paired-end 251bp mode on an Illumina MiSeq (Illumina, San Diego, CA) using a standard MiSeq kit and protocols. Pools were mixed to have 6.5% of the total loaded DNA from the unselected pool, 6.5% of the total loaded DNA from each first-round selected pool, and 3.25% from each second-round selected pool, with 35% Illumina PhiX control DNA to increase diversity and data quality.

### DMS data processing

Raw sequence files were processed into fastq format, split by barcode, allowing up to 1 mismatch, and adapter sequence was removed using Illumina OLB 1.9.4. Split library sequences were processed using scripts from Enrich 0.2 to yield mutation counts in each library. Counts for each sorted library were converted to log2 enrichment relative to the unselected library using custom scripts. An enrichment value was calculated by linear regression of enrichment for each individual substitution at each round. The slope of the regressed line is the enrichment value [44].

### Combinatorial library construction and selection

Twelve positions in HB36.5 that contained substitutions highly enriched against many or all tested subtypes were mutated in a combinatorial library with a total sequence diversity of 10^8^. This library was constructed using recursive PCR assembly, as described below, with the only difference being that the assembly oligos contained degenerate codons designed using GLUE [45] to maximize enriched amino acid codon representation. This library was transformed into yeast using Chao’s method [43] with an efficiency around 10^7^, and sorted by three rounds of yeast display for binding to A/South Carolina/1/1918 HA until sequence convergence was achieved. The final converged sequence, HB36.6, had 9 total mutations relative to HB36.5.

### Recursive PCR assembly

The gene for HB36.6 with 40bp of additional pETFLAG overlap sequence, to allow homologous recombination, was assembled via recursive PCR. Sequences were designed using DNAWorks [46] and purchased from Integrated DNA Technologies, Inc. (Coralville, IA). The outermost two primers were diluted to 5μM and mixed, while the inner primers were diluted to 0.5μM and mixed. A 10μL volume of outer primer mix was added to 12.7μL of inner primer mix, along with 1μL of 10mM DNTPs, 6μL of 5x Phusion buffer, and 0.3μL of Phusion polymerase (NEB, Waltham, MA) for a final volume of 30μL. Product was assembled with 30 rounds of PCR (98°C 30s, 58°C 30s, 72°C 30s). A second round of PCR was used to further amplify correctly assembled product. A 1.25μL aliquot of the first PCR reaction product was added to 5μL of 5x Phusion Buffer, 0.75μL 10mM DNTPs, 2μL of outer primer mix, and 0.25μL of Phusion polymerase in 25μL. The same PCR conditions were used for a further 30 rounds and the product was purified using a QiaQuick PCR cleanup kit (Qiagen, Hilden, Germany) and eluted in EB. Gibson assembly [47] was used to insert the assembled gene into gel-purified pETFLAG vector digested with *NdeI*/*XhoI* (NEB, Waltham, MA). A 1.5μL aliquot of cut vector at 20ng/μL was added to 1μL of assembled gene and 7.5μL of Gibson enzyme mix (all enzymes from NEB, Waltham, MA). The reaction was incubated at 50°C for 1h and 2μL was transformed into 20μL of XL10 Gold chemically competent *E*. *coli* and plated onto a kanamycin agar plate. Plasmid sequences were confirmed by colony PCR and Sanger sequencing, and colonies with correctly assembled plasmid were grown in TB and harvested using a QiaPrep miniprep kit (Qiagen, Hilden, Germany).

### Protein expression and purification

HB36.6 in a pETFLAG vector was expressed in Rosetta2 (DE3) *E*. *coli* cells. HB36.6 used throughout these studies contained the FLAG tag (DYKDDDDK). Cells were grown in 500μL aliquots of medium salt aspartate-glucose (MDG) non-inducing media supplemented with kanamycin at 37°C, 250rpm overnight. Each vial was used to inoculate 500mL of ZYM-5052 auto-induction media [48], which was grown for ~48 hours at 22°C, 250rpm. Cells were harvested by centrifugation and resuspended in 25mL of lysis buffer (50mM Tris, 300mM NaCl, 30mM imidazole, pH 8.2) with half of a dissolved complete, ethylenediaminetetracetic acid (EDTA)-free protease inhibitor tablet (Roche, Basel, Switzerland) and supplemented with DNAse and lysozyme at ~1mg/mL. Resuspended cells were lysed via sonication with a Qsonica Q500 (Fisher Scientific, Hampton, New Hampshire) at 70% power for 10 minutes (20s on/20s off) on ice. Insoluble cell debris was removed by centrifugation for 30m at 40,000g. Supernatant was applied to gravity-flow columns containing 2.5mL of Ni-NTA resin (Qiagen, Hilden, Germany) pre-equilibrated with lysis buffer. Protein was washed with 25mL wash buffer (50mM Tris, 300mM NaCl, 75mM imidazole, pH 8.2) and eluted with 10mL elution buffer (50mM Tris, 300mM NaCl, 300mM imidazole, pH 8.2). Protein was concentrated to ~20mg/mL using a Vivaspin 10kD MWCO centrifugal concentrator (Sartorius Stedim, Goettingen, Germany) at 4000g at 4°C. Imidazole was removed by dialysis (2x 4L buffer) into 50mM Tris, 300mM NaCl, pH 8.2 at 4°C. Concentration was determined by absorbance at 280nm on a NanoDrop spectrophotometer (Thermo Scientific, Waltham, Massachusetts) using extinction coefficients calculated from amino acid sequences. For *in vivo* experiments, proteins were further processed by incubating with magnetic bacterial endotoxin removal beads. Beads were removed with a magnet and then samples were centrifuged to ensure complete removal. Endotoxin lipopolysaccharide (LPS) levels were reduced to <110 EU/mg (Miltenyi Biotec, San Diego, California).

A larger scale purification procedure for HB36.6 was used to produce the protein for the mouse study comparing HB36.6 to oseltamivir. Rosetta2 (DE3) *E*. *coli* cells carrying the HB36.6 pETFLAG vector were cultured in 50 ml of ZY Broth (10 g/l tryptone, 5 g/l yeast extract) with 15 ug/ml kanamycin at 37°C, at 250rpm overnight. The overnight culture was used to seed (3 ml starter culture per bottle) multiple airlift fermentation bottles, each containing 2 liters of autoinduction media ZYP-5052 [48] supplemented with 15 ug/ml kanamycin, and the cultures were sparged with air for 72 hours at 25°C using an airlift LEX Bioreactor System (Harbinger Biotech, Ontario Canada) [49]. Cells were harvested at 4°C from culture media in 2 liter centrifuge buckets, pelleted at 4000g using a Sorvall RC 12 BP centrifuge fitted with an H-12000 swinging-bucket rotor. The masses of the cell pastes were measured to verify proper growth (usually in the range of 20–30 g per 2 liter culture). The pelleted cells were flash frozen in liquid nitrogen and stored at -80°C. Frozen cell pellets from the equivalent of 2 liters of autoinduction culture were thawed by addition of 20 ml of Lysis Buffer (25 mM HEPES pH 7.0, 500 mM NaCl, 5% Glycerol, 0.5% CHAPS, 30 mM Imidazole, 10 mM MgCl2, 1 mM TCEP, 250 ug/ml AEBSF, and 0.025% Azide) and 15 minute incubation at 37°C in a water bath, followed by addition of 0.01g of lysozyme and incubation for an additional 15 minutes in the 37°C water bath. The thawed cell pellet is gently resuspended with a spatula and transferred into a 500 mL beaker inside an ice bath filled with 180 ml of Lysis Buffer. The resuspended pellet is lysed in the ice bath by sonication for 30 minutes at 100W with “10 s ON and 20 s OFF” cycle. After sonication, the crude lysate is clarified with 20ul (25 units/ul) of Benzonase and incubated at room temperature for 40 minutes in 250 ml centrifuge bottles using a Stuart SRT1 rotating mixer. The clarified crude extract is then centrifuged at 14,000 rpm for 1 hour at 4°C using a Sorvall SLA-1500 Rotor and the supernatant is transferred into a clean reservoir. Using an ÄKTAexplorer 100 (GE Healthcare), the supernatant with soluble protein is pumped at 5 ml/min aver a 5 ml Ni-NTA His-Trap FF column (GE Healthcare) pre-equilibrated with Wash Buffer (25 mM HEPES pH 7.0, 500 mM NaCl, 5% Glycerol, 30 mM Imidazole, 1 mM TCEP, and 0.025% Azide). The column is washed with 20 column volumes (CVs) of Wash Buffer and the bound protein is then eluted with 20 ml of Elution Buffer 1 (Wash Buffer + 250 mM imidazole), followed by 20 ml of Elution Buffer 2 (Wash Buffer + 320 mM imidazole). Each imidazole elution pool was then further purified by preparative size exclusion chromatography using an ÄKTAexplorer 100, and a HiLoad 26/60 Superdex 75 preparative-grade column (GE Healthcare) equilibrated with SEC Buffer (25 mM HEPES, 0.5 M NaCl, 5% Glycerol, 1 mM TCEP, pH 7) [50]. Following SDS-PAGE analysis, the peak SEC fractions of HB36.6 were pooled together with additional SEC Buffer such that a HB36.6 was at final concentration of 1–2 mg/ml. Using a Millipore Tangential Flow Filtration System (Millipore Labscale system), the pooled HB36.6 was concentrated to approximately 5 mg/mL using a 5kDa MWCO PES 10cm^2^ membrane, then diafiltered at a constant volume against Storage Buffer (20mM Tris-HCl, 300mM NaCl pH 8) for five diavolumes at 2-8C. Transmembrane pressure was held to 20psi. Concentrated HB36.6 samples were analyzed by SDS-PAGE to verify purity of the samples at >98% and mass spectrometry to verify identity. Endotoxin levels were also measured using the Endosafe PTS reader (Charles River Laboratories), with a result of 17.2 EU per mg of HB36.6. Finally the purified HB36.6 was dispensed into 2 ml vials and flash frozen in liquid nitrogen followed by storage at -80°C.

### Circular dichroism spectroscopy

Purified proteins were tested for folding and denaturation temperature using an Aviv 420 circular dichroism (CD) spectrometer (Aviv Biomedical, Lakewood, NJ). Protein was diluted to 0.6mg/mL in PBS and CD absorbance was measured at 205nm at 25°C. Absorbance was characteristic of a structured α-helical protein. To test the thermal denaturation temperature of HB36.6, absorbance at the 222nm α-helix peak was measured in 2° increments between 15° and 95°C.

### Protease resistance assays

Proteins to be tested were diluted to 1mg/mL in PBS and Gibco 0.25% trypsin/phenol red (Life Technologies, Carlsbad, CA) was diluted to 0.005% in PBS. Equal parts protein solution and trypsin were mixed and incubated at 37°C. Time points were taken by removing 6μL aliquots of solution, mixing with 6μL of 2x SDS loading buffer (100mM Tris, 4% SDS, 0.2% bromphenol blue, 20% glycerol, pH 6.8) and incubating for 2m at 85°C. Denatured aliquots were stored at -20°C until being loaded on a 12% NuPage Bis-Tris gel (Life Technologies, Carlsbad, CA) and run at 150V in 1x MES buffer (50 mM MES, 50 mM Tris Base, 0.1% SDS, 1 mM EDTA, pH 7.3). Negative controls were one lane with undigested protein and one lane with trypsin but no test protein. Gels were scanned and bands were quantified using ImageJ. Band size as a percentage of the undigested negative control was fit by non-linear regression using a custom MATLAB script to derive protein digestion half-lives.

### BioLayer Interferometry (BLI)

Titrations were performed on an OctetRed96 BLI system (ForteBio, Menlo Park, CA) using streptavidin-coated probe tips. Tips were equilibrated for 10m in 50x kinetics buffer, designed to reduce nonspecific binding (PBS, pH 7.4, 0.5% w/v BSA, 0.05% v/v Tween 20) and loaded with 25-40nM biotinylated HA of one of six subtypes in 50x kinetics buffer for 15m. Following a 10m wash and 10m baseline reading in the 50x kinetics buffer, association rates were measured by incubating each tip for 30m in different concentrations of purified HB36.5 or HB36.6 protein spanning the predicted k_D_ for the given HA subtype, diluted in 50x kinetics buffer. Dissociation was measured by then incubating the tips in 50x kinetics buffer for a further 30m. HB36.6 did not show well-defined off-rates, so equilibrium binding constants were computed from the maximum steady-state response reached during the association phase. The limit of detection for the Octet instrument used in these experiments is around 1nM and the equilibrium binding experiments on the Octet yielded values of HB36.5 and HB36.6 against SC1918 at that threshold and within a standard error of each other. However, HB36.6 is a stronger binder against this strain with lower k_off_, though not a different k_on_. Unfortunately, due to equipment limitations, a specific measurement of how much stronger was not possible. The k_D_ values for HB36.6-SC1918 were as low as 1pM but, since the dissociation curves are shallow, any minor deviation would cause enormous changes in the calculated k_D._ Thus equilibrium values are reported. Nonlinear regression curve fitting was done with a custom MATLAB script.

### In vitro antiviral neutralization

MDCK (Madin Darby canine kidney) from American Type Culture Collection (ATCC, Manassas, VA) were grown in Growth medium comprising minimum essential medium (MEM) with non-essential amino acids, 5% FBS and 0.22% NaHCO3. Influenza A/California/07/2009 (H1N1), A/Puerto Rico/08/1934 (H1N1), A/New Caledonia/20/1999 (H1N1), A/Hong Kong/213/2003 (H5N1), A/Nanchang/933/1995 (H3N2), A/Brisbane/10/2007 (H3N2), and A/Hong Kong/33982/2009 (H9N2), were obtained from the Center for Disease Control (Atlanta, GA). Influenza A/Duck/MN/1525/81 was kindly provided by Robert Webster (St. Jude Children’s Research Hospital, Memphis. TN). The viruses were prepared in Madin Darby canine kidney (MDCK) cells, placed in ampules and frozen at -80°C. Cells are seeded to 96-well flat-bottomed tissue culture plates at the proper cell concentration to establish confluent cell monolayers and incubated overnight at 37°C. Various dilutions of test compound were added to each well. Ribavirin (1-β−D-ribofuranosyl-1,2,4-triazole-3-carboxamide), FI6v3, and HB36.6 were tested in half-log increments from 320 μg/ml and below. Virus was added to test compound wells and to virus control wells at about 50–100 cell culture infectious dose per ml. The virus titer was determined by a prior titration, where the most diluted virus stock causes 100% cytopathic effect (CPE) in all wells at the particular virus dilution. Test medium without virus was added to all toxicity control wells and to cell control wells. The plates were incubated at 37°C for 72 hours. Sterile neutral red (0.034% in saline solution) was then added to each well. After two hours at 37°C, all medium was removed and the cells washed with PBS and inverted to drain. Neutral red was extracted from the cells by adding an equal volume mixture of absolute ethanol and Sörensen’s citrate buffer, pH 4.2. The contents of each well are mixed gently and the optical density (O.D.) values of each well are obtained by reading the plates at 540 nm with a microplate reader.

### Negative-stain sample preparation and imaging

Complexes of HA and HB36.6 were prepared for electron microscopy studies by diluting to 2.1 μg/ml in Tris buffered saline and applied to freshly glow discharged carbon coated 400 mesh copper grids for 20 seconds. Two rounds of a 3 μl droplet of 2% uranyl formate were applied and immediately blotted followed by a third 3 μl droplet blotted after 1 minute. Grids were viewed using the FEI Tecnai T12 electron microscope operating at 120 kV accelerating voltage at 52,000 x magnification resulting in a pixel size 2.05 Å at the specimen level. Images were acquired on a Tietz 4k x 4k complementary metal-oxide-semiconductor (CMOS) camera using Leginon [51, 52] MSI-raster 3.0 software package at a defocus of ~1.0 μm. Microscope magnifications were calibrated using a catalase crystal prior to data collection.

### EM data processing and 3D volume reconstruction

Particles were picked automatically using DoG Picker [53] and boxed into 96x96 pixel boxes and aligned using Xmipp CL2D clustering alignment [54]. Ten *ab initio* models of each complex were created using EMAN2CL [55] with C3 symmetry and based on 17 2D class averages of PR8 in complex with HB36.6. Initial models of complexes were then refined against 10,005 raw particles using EMAN [56]. The resolution of the final model was determined to be ~22 Å using an FSC cut-off of 0.5. The UCSF Chimera “Fit in Map” function was used to dock structural models into the EM maps.

### HB36.6 and oseltamivir administration and influenza challenge

Animal studies approved by the University of Washington and Utah State University Institutional Animal Care and Use Committee. Female, 6–8 week-old BALB/c mice were randomly separated in to groups, anesthetized and intranasally administered protein binder (HB36.6) at concentrations varying from 0.01 to 6.0 mg/kg. Two to forty-eight hours later, the mice were anesthetized with 2.5% isoflurane and challenged IN with 3–10 MLD_50_ (fifty percent mouse lethal dose) of A/California/04/09 (H1N1) (CA09), A/PR/8/34 (H1N1) (PR8) or A/Duck/MN/1525/81 (H5N1) (MN81). In a therapeutic setting, mice received the protein binder 0 (2 hours post-infection), +1, +2, +3, or +4 days post infection. The mice were monitored daily for weight loss and survival until 14 days post-infection. Animals that lost more than 30% of their initial body weight were euthanized by carbon dioxide in accordance with our animal protocols. Oseltamivir-treated mice received 2.5 mg/kg of oseltamivir (Roche, Palo Alto, CA) twice daily for 5 days (total of 10 doses) by oral gavage. Oseltamivir was dissolved in water prior to administration. The SCID (Non-Obese Diabetic (NOD), Severe Combined Immunodeficiency (SCID) gamma, strain NOD.Cg-*Prkdcscid Il2rgtm1Wjl*/SzJ) mice and the MyD88-/- (strain *B6*.*129P2(SJL)-Myd88tm1*.*1Defr*/J) mice were purchased from Jackson Laboratory. At least five mice per group were used for each experiment. All mice used for the experiments are included for analyses. For mouse experiments, researchers were not blinded to animal identity.

### Nasal and lung viral titers in mice

Nasal wash samples were collected by making an incision in the trachea and washing the nasal passages with 0.2 ml sterile PBS (pH 7.2). Supernatants from lung homogenates were collected by mincing whole lungs in 500μl MEM media, freeze thawing twice on dry ice, and then centrifuging at 13,000rpm for 10m. The viral titers in the nasal washes and supernatants from lung homogenates were determined using the TCID_50_, as described previously [57]. In brief, monolayers of MDCK cells were inoculated with tenfold serial dilutions of mouse nasal washes in quadruplicate (three total replicates per sample). One hour after inoculation, the supernatants were removed and replaced with MEM media plus antibiotics and 1 μg/ml TPCK-trypsin (Sigma, St. Louis, MO). The viral cytopathic effect was observed for 3 days before viral infectivity in MDCK cells was measured using a hemagglutination assay with 0.33% turkey erythrocytes. The tissue viral titers were calculated using the Reed and Muench method [58] and expressed as log_10_ TCID_50_/g of tissue.

### Enzyme-linked immunosorbent assay (ELISA)

HB36.6-specific IgG antibody levels in mouse serum were assessed by ELISA. Maxisorp (Thermo Scientific-Nunc) were coated with 100 ng/well of HB36.6 in PBS overnight at 4°C. Plates were blocked with 5% nonfat milk powder in PBS for 1h at room temperature, and then washed three times with wash buffer (PBS-T; phosphate-buffered saline containing 0.05% Tween 20). Two-fold serial dilutions of samples were added to the wells and plates were incubated for 1hr at room temperature. Following three washes with PBS-T, plates were incubated with horseradish-peroxidase conjugated goat anti-mouse IgG (1/3,000 dilution) secondary antibodies (Thermo Scientific Pierce) for 1h at room temperature. After five washes with PBS-T, TMB substrate (KPL) was added to the wells for 30 min at room temperature. Color development was stopped by the addition of TMB Stop solution (KPL), and the plates were read at 450nm to measure relative optical densities (O.D.).

### Bio-Plex analysis of cytokine production in lung homogenates

The concentrations of cytokines in lung tissue were measured. On days 2 and 4 post-infection, 8 mice per group were sacrificed and whole lung tissue was collected and immediately frozen. Lungs were thawed, weighed and lysed using the Bio-Plex Cell Lysis Kit (Bio-Rad, Hercules, CA). The levels of interleukin (IL)-6, IL-10, IL-12(p70), interferon (IFN)-γ, and tumor necrosis factor (TNF)-α in the lysate were measured using a Bio-Plex multiplex bead array kit (Bio-Rad, Hercules, CA). The Bio-Plex assay was performed in accordance with the manufacturer’s instructions.

### Histology and immunohistochemistry

During in vivo challenge experiments, lungs were removed from mice and immersed in 10% neutral buffered formalin. Following fixation, tissues were removed from formalin and placed in paraffin. Immunohistochemical staining was performed on the Leica Bond Automated Immunostainer. Sections were deparaffinized in Leica Bond Dewax Solution (Leica Cat No. AR922) and rehydrated through 100% EtOH. After antigen retrieval with EDTA buffer pH 9.0 (Lieca Bond Epitope Retrieval Solution 2, Cat No AR9640) at 100°C for 20m, blocking endogenous peroxidase activity with 3.0% H_2_O_2_ for 5m, and blocking with 10% normal donkey serum in TBS for 20m, the sections were incubated with goat anti-influenza A virus, (Meridian Life Science Inc. Cat No. B65141G) at 1:2000 or normal goat IgG, isotype control, (Invitrogen Cat No. 02–6202) at (1:5000 dilution) both in Bond Primary Antibody Diluent (Leica Cat No. AR9352) for 30m at room temperature. Sections were then incubated with rabbit anti-goat IgG (Jackson ImmunoResearch Cat. No. 305-005-045) 1:1500 + 5% normal donkey serum for 8 minutes at RT followed by incubation with goat anti-rabbit poly-HRP polymer secondary detection (Leica Cat No DS9800) for 8m at room temperature. Sections were then incubated with Leica Bond Mixed Refine DAB substrate detection for 10 minutes at room temperature. (Leica Cat No DS9800). After washing with DIH_2_O, the sections were counter stained with Hematoxylin solution (Leica Bond Refine Kit) dehydrated through 100% EtOH, cleared in Xylene and mounted with synthetic resin mounting medium and 1.5 coverslip.

### Statistical and power analyses

All of the analyses were performed using Graphpad Prism version 5.01. A Student's t test (to compare two samples) and analysis of variance (ANOVA) (to compare multiple samples) were used for statistical analysis. Survival analyses were performed by using the Kaplan-Meier log-rank test. A P value of <0.05 was considered to be significant. For mice, the minimum group size was determined using weight loss data with 100% of control mice becoming infected with CA09. Based on a standard deviation of 2% in weight loss, a group size of n = 5 yields >80% power to detect a minimum of a 10% difference between groups in weight loss using a two-sized t-test with an alpha value of 0.05.

## Supporting Information

S1 Fig Intranasal but not intravenous delivery of HB36.6 protects mice against lethal Influenza virus challenge. (a) Survival and (b) weight change in mice that received 6 mg/kg body weight of HB36.6 intravenously (IV) or intranasally (IN) 2 hours before intranasal challenge with 10 MLD_50_ of CA09 virus. Mean and SEM from n = 5 Balb/c mice per group are shown.
(TIFF)

S2 Fig Intranasal administration of HB36.6 penetrates into the lung. To determine if IN delivery of HB36.6 penetrates into the lower respiratory tract, mice received 6.0 mg/kg of HB36.6 and 6 hours later, lung tissue (blue) was sectioned and stained using anti-FLAG antibodies (brown). Representative images, 10X and 20X (boxed area), from the right lung lobe of the lower respiratory tract from (a) untreated control, (b) HB36.6-treated. Arrows indicate areas of anti-FLAG staining.
(TIFF)

S3 Fig Repeat administration of HB36.6 induces low level antibody does not reduce protection from lethal challenge with CA09. Balb/c mice received 3 intranasal doses of HB36.6 (3.0 mg/kg) spaced two weeks apart and then received a 4^th^ intranasal dose 2 weeks after the 3^rd^ dose and 24 hours prior to lethal challenge with 10 MLD_50_ of CA09 virus. (a) Antibody responses specific for HB36.6 were measured by ELISA in serum collected 2 weeks after each dose of HB36.6 (Doses #1–4). (b) Survival and (**c**) weight change in Balb/c mice following a 4^th^ intranasal dose of HB36.6 and lethal challenge with CA09. Mean and SEM of n = 10 mice per experimental condition are shown.
(TIFF)

S1 Table HB36.6 broadly binds Group 1 HAs. Equilibrium binding constants determined by biolayer interferometry for HB36.6 against six HAs demonstrate broad binding affinity against a variety of Group 1 subtypes.
(DOCX)
