# Supplementary figures and images for: A Computationally Designed Hemagglutinin Stem-Binding Protein Provides In Vivo Protection from Influenza Independent of a Host Immune Response
Source: PLoS Pathog. 2016 Feb 4;12(2):e1005409. doi: 10.1371/journal.ppat.1005409 (PMC4742065; doi:10.1371/journal.ppat.1005409)

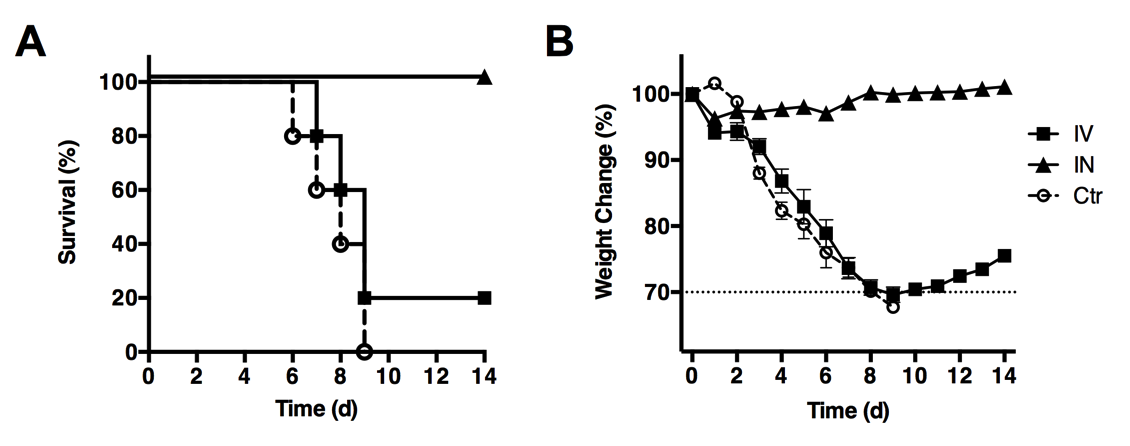

Supplement: S1 Fig — (a) Survival and (b) weight change in mice that received 6 mg/kg body weight of HB36.6 intravenously (IV) or intranasally (IN) 2 hours before intranasal challenge with 10 MLD50 of CA09 virus. Mean and SEM from n = 5 Balb/c mice per group are shown. (TIFF) [file ppat.1005409.s001.tiff]

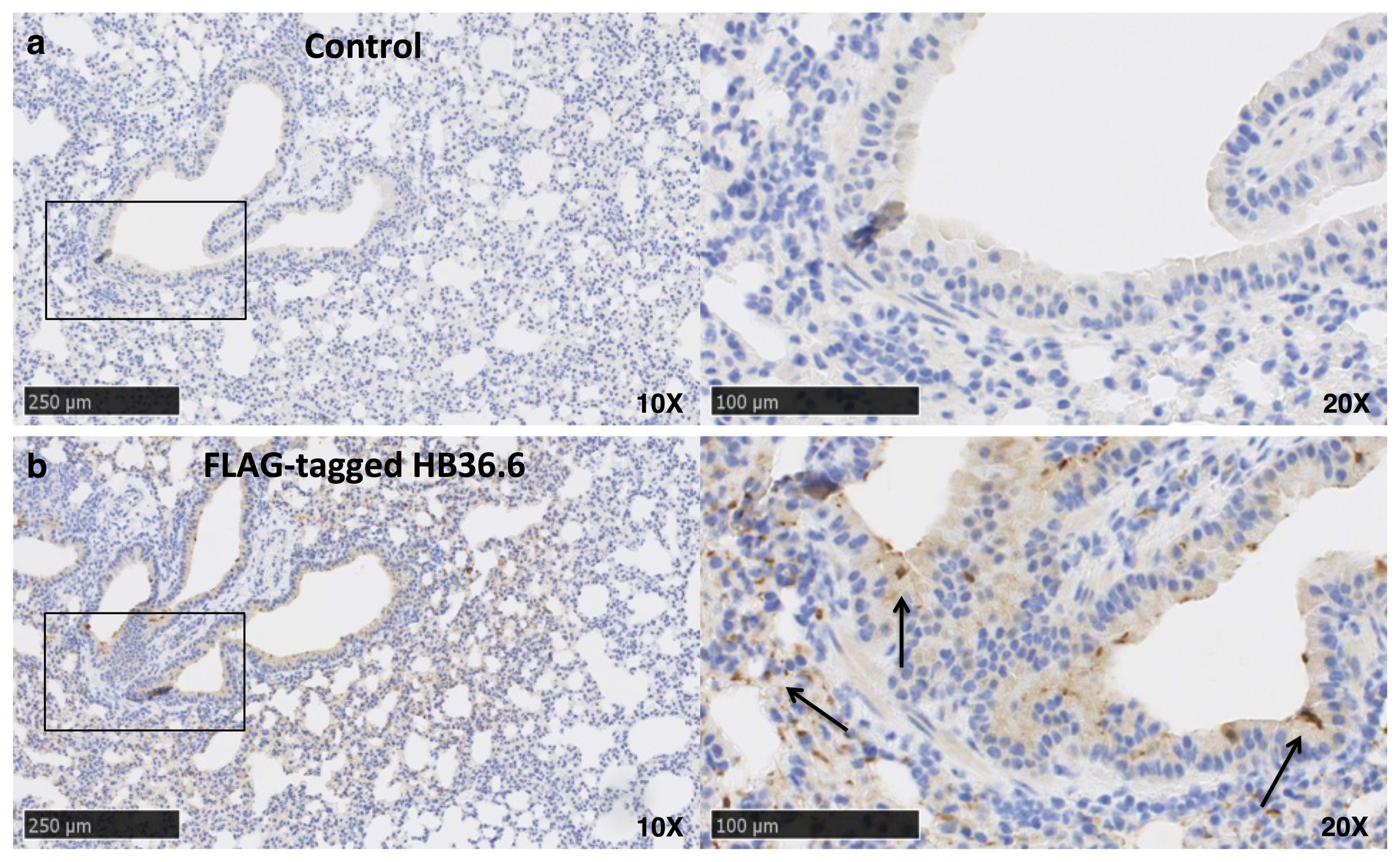

Supplement: S2 Fig — To determine if IN delivery of HB36.6 penetrates into the lower respiratory tract, mice received 6.0 mg/kg of HB36.6 and 6 hours later, lung tissue (blue) was sectioned and stained using anti-FLAG antibodies (brown). Representative images, 10X and 20X (boxed area), from the right lung lobe of the lower respiratory tract from (a) untreated control, (b) HB36.6-treated. Arrows indicate areas of anti-FLAG staining. (TIFF) [file ppat.1005409.s002.tiff]

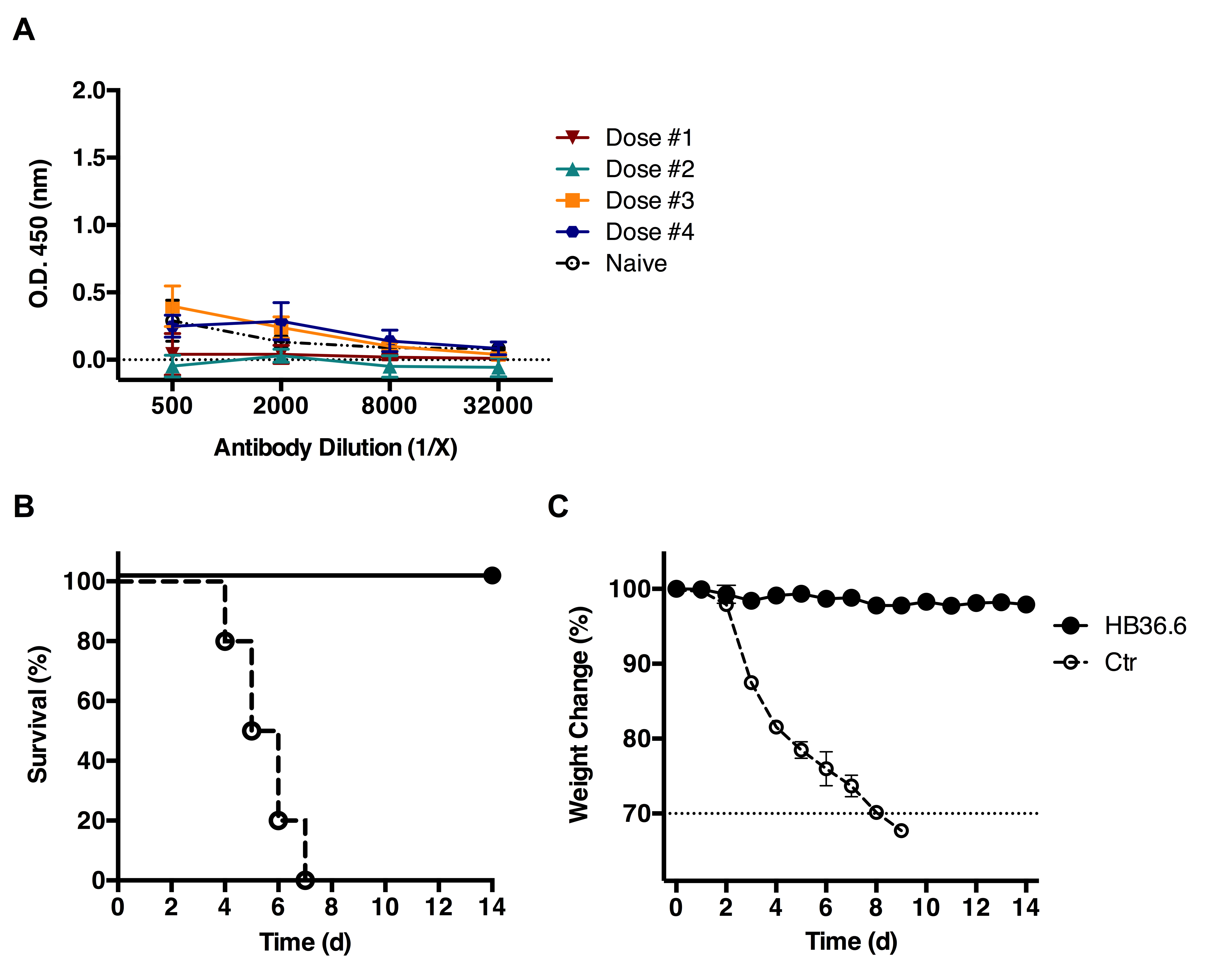

Supplement: S3 Fig — Balb/c mice received 3 intranasal doses of HB36.6 (3.0 mg/kg) spaced two weeks apart and then received a 4th intranasal dose 2 weeks after the 3rd dose and 24 hours prior to lethal challenge with 10 MLD50 of CA09 virus. (a) Antibody responses specific for HB36.6 were measured by ELISA in serum collected 2 weeks after each dose of HB36.6 (Doses #1–4). (b) Survival and (c) weight change in Balb/c mice following a 4th intranasal dose of HB36.6 and lethal challenge with CA09. Mean and SEM of n = 10 mice per experimental condition are shown. (TIFF) [file ppat.1005409.s003.tiff]
