# Supplementary material for: A Computationally Designed Hemagglutinin Stem-Binding Protein Provides In Vivo Protection from Influenza Independent of a Host Immune Response
Source: PLoS Pathog. 2016 Feb 4;12(2):e1005409. doi: 10.1371/journal.ppat.1005409 (PMC4742065; doi:10.1371/journal.ppat.1005409)
Supplement: S1 Table — Equilibrium binding constants determined by biolayer interferometry for HB36.6 against six HAs demonstrate broad binding affinity against a variety of Group 1 subtypes. (DOCX) [file ppat.1005409.s004.docx]

| **Influenza Virus** | **k_D_ (nM)** |
| --- | --- |
| A/Puerto Rico/8/1934 (H1N1) | 16 |
| A/New Caledonia/20/1999 (H1N1) | 3 |
| A/Japan/305/1957 (H2N2) | 52 |
| A/turkey/Massachusetts/3740/1965 (H6N2) | 16 |
| A/duck/England/1/1956 (H11N6) | 60 |
| A/gull/Maryland/704/1977 (H13N6) | 161 |
